# Glut1 and Glut3 as Potential Prognostic Markers for Oral Squamous Cell Carcinoma

**DOI:** 10.3390/molecules15042374

**Published:** 2010-04-01

**Authors:** Fernanda Rocha Rojas Ayala, Rafael Malagoli Rocha, Kátia Cândido Carvalho, André Lopes Carvalho, Isabela Werneck da Cunha, Silvia Vanessa Lourenço, Fernando Augusto Soares

**Affiliations:** 1 Department of Anatomic Pathology, Hospital A.C Camargo, São Paulo, Rua Antonio Prudente, 109–1 andar, CEP: 01509-010, São Paulo, SP, Brazil; 2 Department of Head and Neck, Hospital A.C Camargo, São Paulo, SP, Brazil; 3 Department of Head and Neck, Hospital do Cancer, Barretos, SP, Brazil; 4 Department of General Pathology, Dental School, University of São Paulo, SP, Brazil

**Keywords:** oral carcinoma, GLUT1, GLUT3, immunohistochemistry

## Abstract

We associated clinical-pathological features of 142 OSCC with the expression pattern of GLUT1 and GLUT3 in order to estimate their prognostic value. **Methods:** Clinical-pathological features and overall survival data of 142 patients with Oral Squamous Cell Carcinoma (OSCC) were retrospectively reviewed from A.C.Camargo hospital records. A tissue microarray (TMA) was built for the immunohistochemical (IHC) analysis of GLUT 1 and GLUT 3. IHC results were evaluated according to the staining pattern and number of positive cells. **Results:** GLUT 1 was over expressed in 50.3% of OSSC cases showing membrane staining pattern. However, nuclear expression was observed in 49.7% of the analyzed cases. GLUT 3 over expression was detected in 21.1% of OSCC cases. The pattern of GLUT 1 expression showed significant association with alcohol consumption (p = 0.004). Positive cell membrane GLUT 3 protein expression was associated with advanced clinic-staging of tumours (p = 0.005) as well as with vascular embolization (p = 0.005). Positive expression of GLUT 3 was associated with unfavorable free-disease survival (p = 0.021). **Conclusion:** GLUT1 and GLUT3 protein expression evaluated by immunohistochemistry are, significantly, indicators of poor prognosis outcome in oral squamous cell carcinoma, probably due to the enhanced glycolytic metabolism of more aggressive neoplastic cells.

## 1. Introduction

Increased glycolytic metabolism is a characteristic of malignant cells, also many mechanisms seem to contribute to the glycolytic phenotype both in normal and neoplastic cells. Since then, increased and deregulated expression of glucose transporter protein, such as GLUT1 and GLUT 3, has been associated with malignancy [1].

Some studies indicated that patients with non-small cell lung carcinoma [2], colorectal carcinoma [3], and gastric carcinoma [4], showed expression of GLUT1 and were associated with enhanced tumor aggressiveness and poor survival. Consequently, a large variety of other tumors has been related to GLUT1 and GLUT3 over expression, such as pancreatic carcinomas [5,6,7], cervical carcinoma [8], esophageal squamous cell carcinoma [9], and squamous cell carcinoma of the head and neck (SCCHN) [10,11,12]. 

Although GLUT1 expression is a common feature in patients with head and neck squamous cell carcinoma (HNSCC), the prognostic value of this parameter has not been analyzed systematically for this tumor type. To the best of our knowledge, only Baer and co-workers considered the prognostic significance of GLUT1 in HNSCC [5]. Additionally, the significance of GLUT1 and GLUT3 expression in oral carcinomas remains unclear and a large amount of contradictory results can be found in the literature. Based on that, our study has given further evidences of the relevant value of the glucose transporter proteins in Oral Squamous Cell Carcinoma (OSCC) evaluating GLUT1 and GLUT3 expressions using immunohistochemical techniques and correlating this expression with clinical data of patients. 

## 2. Results and Discussion

### 2.1. Patients’ Clinical Data

The cohort of OSCC cases of this study included 112 male and 30 female patients (for a total of 142). The mean age of the patients was 56.6 years (range, 30–90 years) and the median was 57 years. The follow-up period of this group of patients ranged from 1 day to 157.6 months with a median of 64.9 months. 

The sites of the primary tumors were, in order of frequency, as follows: tongue (54 cases or 38%), floor of mouth (42 cases or 29.6%), retromolar region (19 cases or 13.4%), mandibular gingiva (14 cases or 9.9%), maxillary gingiva (six cases or 4.2%), buccal mucosa (six cases or 4.2%) and palate (one case or 0.7%). Clinical stage of tumors included: Stage I/II, 26.1% (n = 37); Stage III/IV, 73.9% (n = 105). Seventy patients (49.3%) confirmed lymph nodes involvement. Disease outcomes recorded showed 43% of patients were alive without disease; 31.7%, died through disease complications; 18.3% died for reasons unrelated to the disease; and 1.4%, were alive with disease. Information was not available for 5.6% of cases. A summary of this data is provided in Table 1.

### 2.2. GLUT1 and GLUT3 Immunohistochemical Expression

**Figure 1:**
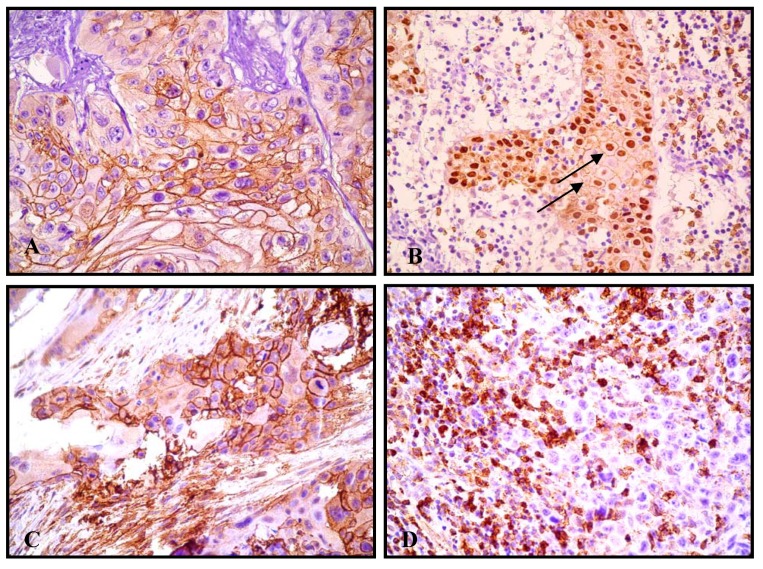
A) GLUT1 membrane immunostaining in oral squamous cell carcinoma, 400×; B) GLUT1 expression in oral squamous cell carcinoma. Note both GLUT1 expression: membrane and nucleus (arrows), 200×; C) GLUT3 membrane immunostaining in oral squamous cell carcinoma; D) No GLUT3 immunostaining. Note also GLUT3 expression in the inflammatory cells, 200×.

Immunohistochemical analyses showed GLUT1 expression in 135/142 cases, with clearly stronger and more extensive staining in neoplastic tissue than in normal adjacent hyperplastic epithelia. In regarding to GLUT1 protein pattern, a membrane pattern was seen in 68 of 135 (50.3%), whereas a nuclear expression appeared in 67 of 135 cases (49.7%, Figure 1). 

According to the number of positive cells, GLUT1 partial expression was observed in 58 cases (40.8%), while its total frequency expression was seen in 77 cases (54.2%). Interestingly, GLUT1 expression was observed in the majority of poorly differentiated cases, which showed an “antistromal” pattern - enhanced staining in central and perinecrotic zones. Conversely, well-differentiated tumors showed an absent GLUT1 staining in the central zones of the tumor, with its expression localized in the peripheral neoplastic area. 

**Figure 2:**
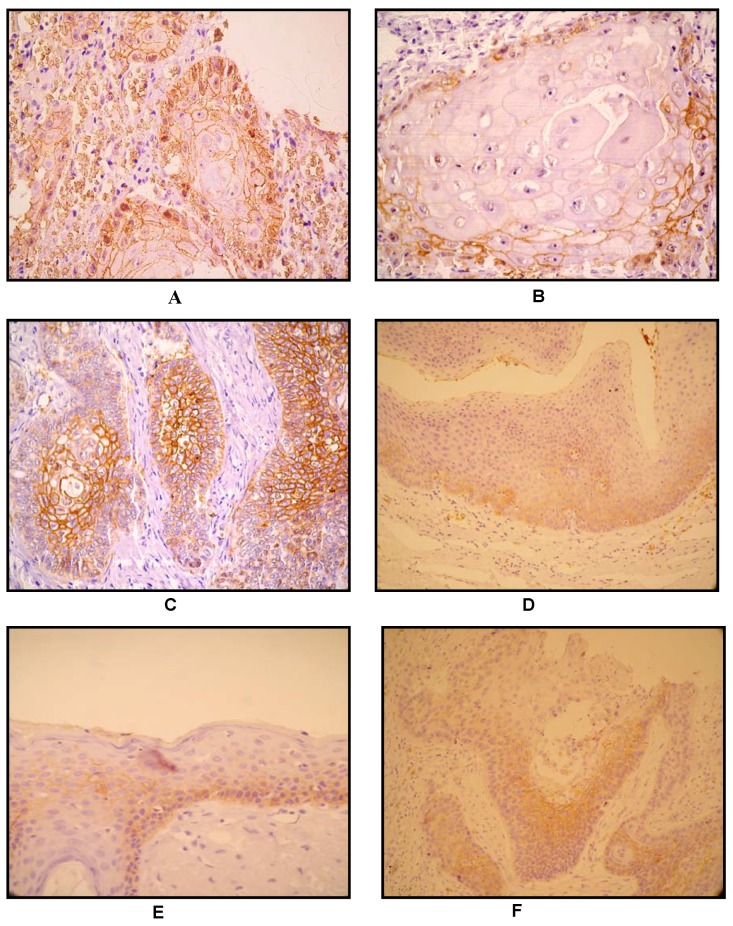
A) Keratinizing squamous carcinoma. GLUT1, prostromal staining pattern. Note positivity at periphery of tumour nest, in non-keratinizing basaloid cells, and loss of GLUT1 expression accompanying keratinization at the centre of tumour nest, 200×; B) 400×; C) Non - keratinizing poorly differentiated carcinoma, 100×. In the absence of squamous differentiation/keratinization: GLUT1 displays an antistromal pattern, suggestive of hipoxia-driven GLUT1 induction. D) and E) Basal staning pattern with the superficial layers showing little or no GLUT1 staining in the normal epithelium; F) GLUT1 immunostain (arrow). Squamous intraepithelial neoplasia, non- staining of basal layer.

On the other hand, in non-neoplastic squamous epithelium, GLUT1 staining was usually undetectable or was weakly detected in supra-basal layers. Thus, a range of staining patterns was observed within the epithelia; in this manner, many cases showed a predominantly basal staining with a superficial layer showing either little or no GLUT1 staining (Figure 2).

Alternatively, GLUT3 expression that was evaluated in all 142 cases showed expression in 30 cases (21.1%), while 112 cases (78.9%) was negative. Notably, a strong GLUT3 immunoexpression was observed in all inflammatory cells adjacent to neoplastic islands, which were considered as an internal control (Figure 1).

### 2.3. GLUT 1 and GLUT 3: Immunoexpression and Clinical-Pathological Parameters

Univariate statistical analysis showed a significant association of cases with a reported history of alcohol consumption with GLUT1 staining nuclear pattern (p = 0.042). In relation to the GLUT1 frequency of cells stained, statistically significant results were found with: gender (p = 0.001), alcohol consumption (p = 0.030) and clinic tumor stage (p = 0.028) (Table 2). In respect to GLUT3 analysis, its expression showed a significant relationship with clinic tumor stage (p = 0,005) and to vascular embolization (p = 0.005) (Table 3).

It was noteworthy that for both proteins no significant association was found with primary site and lymph node involvement. Yet, no significant statistical relationship was found between GLUT 1 and GLUT 3 expression at OSCC tumors.

**Figure 3:**
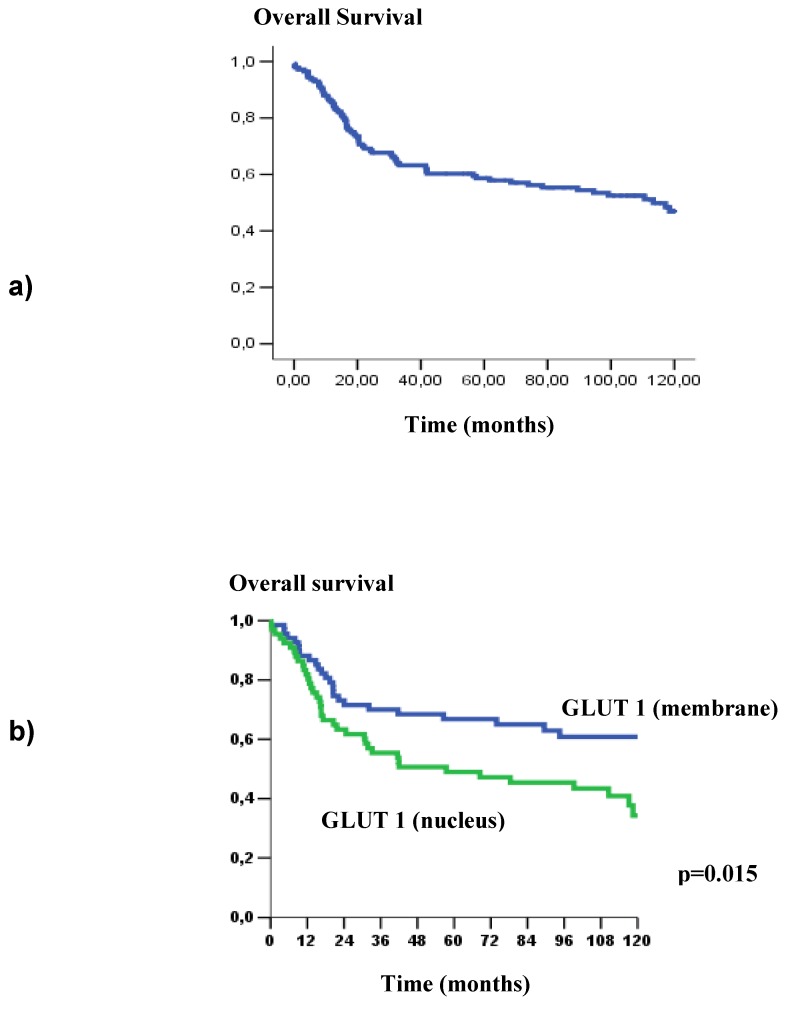

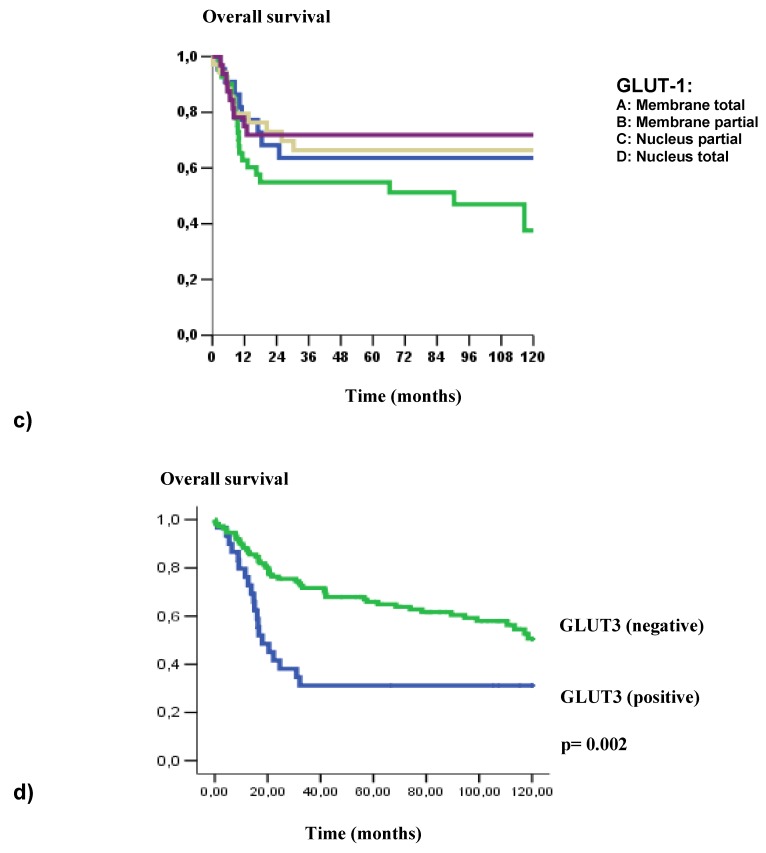
a) Kaplan –Meier curve of overall survival at five years in patients with OSCC (52.1%); b) Overall survival as a function of GLUT1 staining pattern (*p* = 0.015); c) Overall survival as a function of GLUT1 frequency, according to stratified cells (*p* = 0.041); d) Overall survival as a function of GLUT3 staining pattern (negative or positive) (*p* = 0.002).

**Figure 4:**
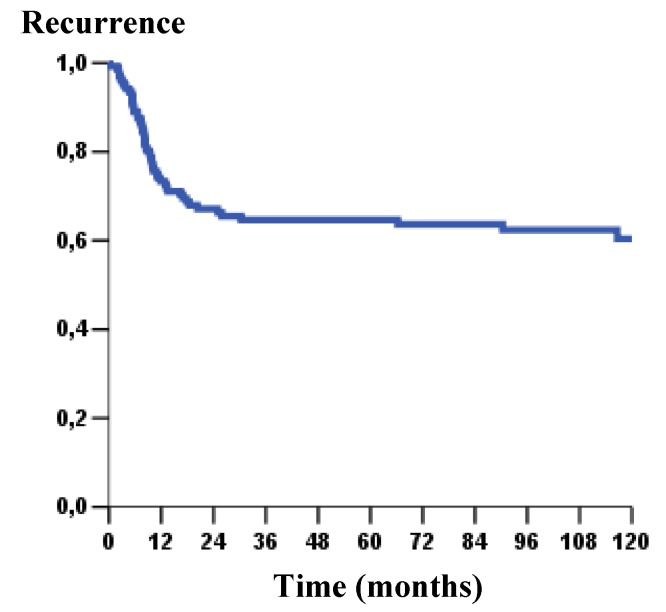
Kaplan–Meier curve of recurrence-free survival in 5 year in OSCC (64.8%).

**Figure 5:**
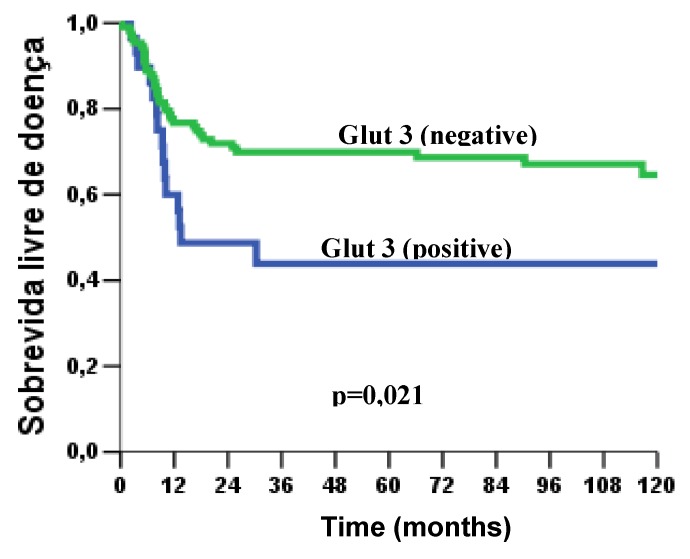
Kaplan – Meier curve of disease - free survival as a function of GLUT3 staining pattern (*p* = 0.021).

Concerning to these patients’ outcome, poor survival rate was significantly associated with the GLUT1 nuclear expression (p = 0.015) (Figure 3). Likewise, GLUT3 expression showed a strong impact on overall survival (p = 0.002), poorer survival curves were verified in patients that showed GLUT3 expression (Figure 3). At the same way, in disease-free survival curves, we observed a statistical difference with GLUT3 expression (p = 0.021); clearly, patients that showed presence of GLUT3 revealed higher risk of recurrence (Figure 4 and Figure 5).

To a great extent, multivariate analysis confirmed GLUT1 expression, GLUT3 expression and vascular embolization as significant markers of independent prognostics for overall survival. Also, the clinic tumor stage was maintained as an independent prognostic marker for disease-free survival. The following hazard ratios were assigned: high GLUT1 expression, 2.066, (p = 0.006); GLUT3 expression, 1.933; p = 0.018); vascular embolization, 2.165, p = 0.002 and clinic stage, 3.304, (p = 0.006) (Table 4 and Table 5).

The enhanced expression of GLUTs proteins and the increased glucose metabolism have been previously investigated in a variety of tumors; however, poorly is known about these proteins in OSCC. Our cohort of 142 cases showed significant associations of GLUT1 and GLUT3 with clinical-pathological features of OSCC patients, which led to the light of their importance as prognostic value in this tumor type. In addition, GLUT1 protein expression confirmed to be a common characteristic of OSCC, whilst GLUT 3 expression was detected only in a limited number of specimens. 

The glucose transporters proteins, GLUT 1 and GLUT 3, largely mediate basal glucose transport in cancer cells, facilitating the maintenance of glycolytic energy metabolism in cases of limited supply of the substrate in moderately to poorly perfused regions [1]. Also, the expression of these proteins might reflect the activity of other oncogenic pathways independent of hypoxia [13]. 

From the aforementioned results, the GLUT1 staining was more evident in the central neoplastic nests as well as around necrotic areas. This distant localization from the supply of blood vessels naturally reinforces the concept of the existence of hypoxia-induced GLUT1 [14,15,16]. 

Also, our findings concur with the Reisser *et al.* [17] study, which demonstrated the presence of GLUT1 in the outer layers of tumor nests, attributing this staining pattern to differentiation functions of this protein, which in intraepithelial lesions was predictive of the differentiation status associated with invasive carcinoma [7]. 

In regarding to protein staining pattern, GLUT1 nuclear expression was associated with parameters of poor prognosis, supported by other study [4], this might be an additional clue to understanding the importance of this protein, not only in glucose transport, but also in other cascades of the cell machinery.

In contrast to GLUT1 expression, the GLUT3 protein was positive in only 30/142 cases (21.1%). However, we observed a strong GLUT3 expression in all inflammatory cells adjacent to the tumor. Mochizuki *et al*. [8], also found GLUT1 and GLUT3 highly expressed in inflammatory tissues, besides malignant tumors. Yet, this study showed that while GLUT1 expression was significantly more frequent in tumor tissue than in inflammatory tissue, while GLUT3 expression tended to be higher in inflammatory lesions. This finding may reflect the involvement of GLUT3 in the earlier stages of carcinogenesis of some tumors. 

As referred to clinical parameters, our results showed that GLUT1 pattern expressions was associated to alcohol consumption, advanced tumor staging and nodal metastasis, which corroborated with the clinical parameters observed in other malignancies such as esophageal carcinomas [17,18], suggesting a connection of GLUT1 with poor prognosis. 

Hence, from our records, in conformity with other studies, lead us to believe that GLUT3 expression may be related to clinic stage of OSCC patients (p = 0.005) [5,16]. 

Furthermore, our multivariate analysis findings confirmed a relationship of both proteins to patients that presented low survival rates, signifying that GLUT1 and GLUT3 may indicate a background of poor prognosis. This relationship showed consistency with studies in laryngeal carcinomas that emphasized the importance of GLUT3 as a predictor of poor prognosis, but no value for GLUT1 as a prognostic indicator [5]. However, GLUT1, which is invariably expressed in head and neck tumors, demonstrated the relationship between intense GLUT1 expression in OSCC with the presence of regional metastasis and disease-related death [14]. 

## 3. Experimental

### 3.1. Patients

One hundred and forty two patients diagnosed with OSCC were selected for the study. All these patients underwent surgical resection in the Head and Neck Department of A.C. Camargo Hospital of São Paulo, Brazil. Clinical information was retrospectively gleaned from patients’ records including demographic data, surgical resection and clinical outcome. Each case was reanalyzed and diagnoses were confirmed. H&E slides were evaluated in order to select representative tumor areas for the TMA construction.

### 3.2. Tissue microarray construction (TMA)

Areas to be used for the TMA construction were selected on the HE slide and on the correspondent donor block of each case. The tissues corresponding to selected areas were sampled using a manual arraying instrument (Manual Tissue Arrayer 1, Beecher Instruments, Sun Prairie, WI, USA). The sampling consisted of two cores from different areas of the tumor, placed coordinately in the array. Separate samples of normal oral mucosa were used as controls. After the arraying was completed, TMA blocks were sectioned at the thickness of 4 μm. One section was stained with hematoxylin and eosin to check for the presence of neoplastic structures, and two other slides, distant 40 μm from each other were used for the immunohistochemical study. 

### 3.3. Immunohistochemistry

Four μm sections from the TMA containing 142 OSCC specimens were re-hydrated and incubated in citrate buffer, pH 6.0 in a pressure pan for antigen retrieval. Incubation in 3% aqueous hydrogen peroxide for 30 min followed to quench endogenous peroxidase activity. Incubation with 1% bovine serum albumin (BSA) and 5% fetal calf serum (FCS) in Tris-HCl pH 7.4 for 60 min at room temperature was performed to suppress non-specific binding of subsequent reagents. The sections were then incubated with the polyclonal rabbit anti-human GLUT1 antibody (DAKO, Carpenteria, CA, USA) used at 1/500, and with polyclonal rabbit anti-human GLUT3 antibody (Lab Vision Corporation, Fremont, CA, USA) at 1/400, for 2 h at room temperature. The reaction followed with incubation of the sections with the Advance Kit (DAKO) (ROCHA *et al.,* 2009). Staining was completed by incubation with 3,3-diaminobenzidine tetrachloride (DAB) for 3 min. The sections were then lightly counterstained with Mayer’s hematoxylin, dehydrated and mounted with glass coverslip and xylene based medium.

Negative controls were obtained by incubating the sections with non-immune serum. Erythrocytes, which were present in every section, served as internal controls for GLUT1 and inflammatory cells of adjacent tumor were used as internal controls for GLUT3. All immunohistochemical reactions were carried out in duplicates. Two pathologists analyzed the results simultaneously using a conventional optical multihead microscope equipped with a digital camera for photographic registration. Disagreements were assessed by consulting a third expert pathologist (IWC).

### 3.4. Evaluation of stained sections: GLUT1 and GLUT3

Cellular patterns of staining and the number of positive cells were recorded for every core included in the OSCC TMA. For GLUT1, we considered positive those cases in which at least 10% of the membrane and nucleus were stained moderate. The positivity was further evaluated according to the area stained: totally positive (more than 85% of neoplastic cells stained) or partial positive (less than 85% of neoplastic cells stained). For GLUT3, only the presence or absence of moderate protein expression on the membrane of at least 10% of tumor cells was considered positive. For statistical analysis, the results obtained in the microscopic analysis were grouped in two categories: M - membrane and N – nucleus. 

### 3.5. Statistical analysis

All statistical analysis were performed using the SPSS 13.0 statistical software program (SPSS, Chicago, IL). The chi - square test and Fisher’s exact test were used to analyze the association between clinical-pathological parameters and molecular biomarker immunoexpression. The five-year survival rates were estimated using the Kaplan-Meier´s method, and the log-rank test was used to compare the curves. Cox proportional hazards model was performed to find independently risk factors for death. For all tests, alpha error was set at 5%.

## 4. Conclusions

Taken all together, GLUT1 and GLUT3 protein expression evaluated by immunohistochemistry appear to define a small clinic subset of OSCC patients with distinctly poor outcome. The enhanced glycolytic metabolism of these proteins at more aggressive tumor cells indicates potential prognostic value in stratifying patients for risk.

## Figures and Tables

**Table 1:** Clinico-pathological information of OSCC patients.

Varible	Group	Cases (%)
**Gender** **(n = 142)**	Male Female	112 (78.9) 30 (21.1)
**Race** **(n = 142)**	Caucasians Others	122 (85.9) 20 (14.1)
**Tabacco** **(n = 142)**	No Light Moderate/Severe Ignored	15 (10.6) 99 (69.7) 17 (12) 11 (7.7)
**Alcohol** **(n = 142)**	No Light Moderate/Severe Ignored	27 (19.0) 89 (62.7) 15 (10.6) 11 (7.7)
**Site** **(n = 142)**	Tongue Gingiva Floor of the mouth Palate Jugal Retromolar	54 (38) 20 (14.1) 42 (29.6) 1 (0.7) 6 (4.2) 19 (13.4)
**Vascular Invision** **(n = 142)**	No observed Yes Ignored	75 (52.8) 53 (37.3) 14 (9.9)
**Clinic Stage** **(n = 142)**	I/II III/IV	37 (26.1) 105 (73.9)
**Recurrence** **(n = 142)**	No Yes	92 (64.8) 50 (35.2)
**Site of Recurrence** **(n = 142)**	Local Others Local/others No recurrence	41 (28.8) 7 (4.9) 2 (1.4) 92 (64.8)
***Status* of last contact** **(n = 142)**	Alive wth disease Alive wthout disease Death due to disease Death due to other causes Lost	2 (1.4) 61 (43) 45 (31.7) 26 (18.3) 8 (5.6)

**Table 2:** Association between GLUT1 expression and clinic-pathological feature in OSCC.

Variable (n = 135)	Category	GLUT-1/grouping				
		M	MN/N OR N	p
**Gender**	Male Female	53(39.3) 15(11.1)	54(40) 13(9.6)	0.832
**Alcohol**	No Light Moderate/severe	14(11.3) 37(29.8) 11(8.9)	11(8.9) 48(38.7) 3(2.4)	0.042
**Staging**	I/II III/IV	19(14.1) 49(36.3)	16(11.9) 51(37.8)	0.695
**Vascular Embolization**	No yes	31(25.6) 29(24)	38(31.4) 23(19)	0.273

M- Membranaee; MN/N: Membranaee nucleus /nucleus; p: value.

**Table 3:** Association between GLUT3 expression and clinic-pathological feature in OSCC.

Variable (n = 142)	Category	GLUT-3		
		Positivo(%)	Negativo(%)	p
Gender	Male Female	87(61.3) 25(17.6)	25(17.6) 5(3.5)	0.619
Alcohol	No Light Moderate/severe	3(2.3) 22(16.8) 3(2.3)	24(18.3) 67(51.1) 12(9.2)	0.316
Tabacco	No Light Moderate/severe	1(0.8) 24(18.3) 3(2.3)	14(10.7) 75(57.3) 14(10.7)	0.279
Staging	I/II III/IV	2(1,4) 28(19,7)	35(24.6) 77(54.2)	0.005
Vascular Embolization	No yes	10(7,8) 19(14,8)	65(50.8) 34(26.6)	0.005

p: p value.

**Table 4:** Multivariate analysis by Cox regression model: Overall survival.

Variable	Category	HR (95% IC)	p valor
Vascular embolization	No	1.0 (ref.)	
	Yes	2.165 (1.295–3.618)	0.003
GLUT-1	(group)	1.0(ref.)	
GLUT-1/pattern	(group 1)	2.066( 1.232–3.464)	0.006
GLUT-3	Negative	1.0 (ref.)	
	Positive	1.933(1.119–3.339)	0.018

GLUT-1/ pattern; M- Membranaee; MN/N: Membranaee - nucleus/ nucleus.

**Table 5:** Multivariate analysis by Cox regression model: Overall survival: Disease-free survival.

Variable	Category	HR (95% IC)	p valor
staging			
	I/II	1.0 (ref.)	
	III/IV	3.304(1.407–7.762)	**0.006**
